# Four dimensional material movies: High speed phase-contrast tomography by backprojection along dynamically curved paths

**DOI:** 10.1038/s41598-017-06333-6

**Published:** 2017-07-26

**Authors:** A. Ruhlandt, M. Töpperwien, M. Krenkel, R. Mokso, T. Salditt

**Affiliations:** 1 Institut für Röntgenphysik, Universität Göttingen, Friedrich-Hund-Platz 1, Göttingen, Germany; 2 Swiss Light Source, Paul Scherrer Institute, 5232 Villigen, Switzerland

## Abstract

We present an approach towards four dimensional (4d) movies of materials, showing dynamic processes within the entire 3d structure. The method is based on tomographic reconstruction on dynamically curved paths using a motion model estimated by optical flow techniques, considerably reducing the typical motion artefacts of dynamic tomography. At the same time we exploit x-ray phase contrast based on free propagation to enhance the signal from micron scale structure recorded with illumination times down to a millisecond (ms). The concept is demonstrated by observing the burning process of a match stick in 4d, using high speed synchrotron phase contrast x-ray tomography recordings. The resulting movies reveal the structural changes of the wood cells during the combustion.

## Introduction

In order to understand and model materials, knowledge about the structure has to be complemented by knowledge about dynamics and functional processes. In general, it is necessary to determine the three-dimensional (3d) structure as a function of time. Ideally, a four-dimensional (4d) ‘material movie’ would allow to observe the dynamics of the assembly and disassembly of structure at the required spatial and temporal resolution. A chemical reaction is a perfect example. Both for a fundamental understanding of reactions in disordered systems, as well as for applications of heterogeneous catalysis, we need to know how a material changes during the course of a reaction. This is experimentally challenging, since coupled reaction and diffusion processes require the full spatiotemporal domain. In order to track down turnover rate, reaction products and the evolving material structure, the dynamics has to be assessed within the entire volume, not only by projections or surface observations. However, 4d movies of a material during a chemical reaction, even a simple combustion, are almost non-existent. Optical methods are often hampered by smoke and gas convection, even if the material is not opaque per se.

As an emerging field, time-resolved x-ray computed tomography (CT) or dynamic tomography (dynamic CT) has a unique potential for non-invasive analysis of materials, combining high spatial with temporal resolution. Novel experimental capabilities have now been opened up by highly brilliant synchrotron sources, new contrast mechanisms including phase contrast, as well as faster detectors and progress in data acquisition. This opportunity for 4d material movies, however, can only be fully exploited if recording schemes and reconstruction algorithms are generalized from the conventional static case to meet the challenges of *dynamic* CT.

Usually, for revealing a static 3d structure, the object is rotated and many projections over a range of 180° have to be acquired. The minimum time required for a complete scan is influenced by multiple factors: photon flux, camera efficiency and repetition rate, as well as the rotation speed. Due to radiation damage, even if available, the flux on the sample cannot be increased without limit in order to cut down the acquisition time. Further, the rotation speed cannot be increased arbitrarily. For example, a single image should not be blurred by rotation. Hence, the recording of the full 180° range will often be too slow to justify the assumption of static conditions in tomography.

**Figure 1:**
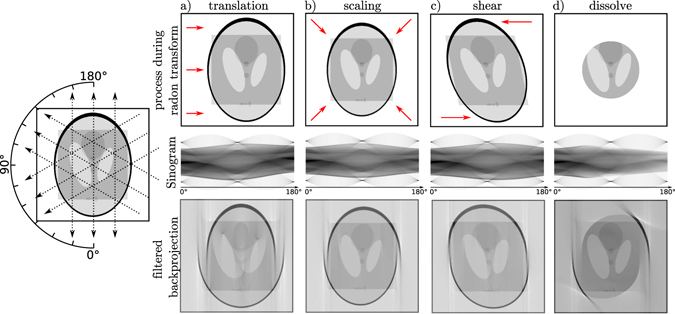
Illustration of artefacts induced by motion and dynamic processes during a tomographic measurement. The starting point *t* = 0 for each simulated measurement was the image depicted on the left side with a size of 512^2^ pixels. Notice that the black frame is a part of the object but only the inner Shepp-Logan phantom and the rectangle were modified while the object was projected from 0° to 180° at a constant rate. The inner part was (**a**) linearly translated, (**b**) scaled with a linear decrease of the scaling factor, (**c**) sheared linearly and (**d**) ‘dissolved’ by multiplying a mask with linearly shrinking radius. The images in the upper row show the maximum change at the end of the tomographic scan. Each of the corresponding sinograms depicted in the middle row consists of 804 equidistant projections to satisfy angular sampling. Common filtered backprojections of the sinogram data are depicted in the bottom row. They show pronounced artefacts originating from the motion, like false shapes, missing features and non-closed circles. Vertical ‘streaks’ in the 0° and 180° direction are clearly visible in each reconstruction, since temporal and thus structural differences are largest for the corresponding projections.

As a consequence, the data will be inconsistent with respect to the paradigm of static tomography. Direct reconstructions result in distorted objects with considerable artefacts like those shown in Fig. 1 for simulated simple object motions during the acquisition time. This observation is the starting point of several approaches to 4d tomography, including optimized weighting schemes, corrections for affine motion, local time-stationarity hypothesis, and periodicity hypothesis (see ref.^1^ for a review). Notably, for the special case of cyclic processes such as a beating heart or a breathing lung, acquisitions can be gated or triggered to cover different phases of the motion^1–3^. If all the projections are captured at the same state of the motion or even a whole cycle is recorded for every angle, static solutions can be generated for the different times within one cycle. Hardware gating as well as software gating (*a posteriori*) have both found convincing implementations, unraveling complex spatiotemporal processes such as the muscle movement during insect flight^4,5^. However, such approaches fail for non-cyclic processes. In this case spatiotemporal reconstruction from the – as such – inconsistent projections is much more challenging. Sophisticated regularized approaches exploit for example sparsity in the time evolution^6^, but some kind of prior information and/or special sampling are required^6–8^. To this end, projection angle sequences with large angular increments are best suited to estimate the spatiotemporal evolution^6,9^. Features of the object can then for example be triangulated from almost orthogonal directions, recorded at minimum time difference. A guess for the object can then be transformed in a way to best fit a sparse set of projections. This is for example exploited in high speed magnetic resonance tomography (MRT), where the projection angles can be freely chosen by selection of the magnetic force gradients^9^. Contrarily, micro-CT is typically based on a single x-ray source, which cannot be rotated freely (synchrotron). Hence, the object has to be rotated, which limits the choice of consecutive projections angles. To date, direct reconstruction methods of such data are mostly restricted to global affine transformations since straight lines remain straight lines after transformation and the motion can be estimated and corrected on the projections^10^. Unfortunately, those approaches are not applicable even to the simple examples in Fig. 1(a–c), where the black border remains static during the transformation of its content. However, the approach points into an interesting direction. If the motion is known, it can be possible to reconstruct consistent 3d representations from the inconsistent projections. Tomographic reconstruction with known motion has been investigated in the literature^1,10–12^ and schemes have shown success in the context of medical CT (heart beat, breathing)^13–17^, however, either for pre-determined motion or for rather sparse objects and over-sampled images. Typically, in such applications the resolution is much smaller than the voxel size, and image artefacts are more deteriorating than loss of resolution.

Here, we present a method addressing the requirements of dynamic CT for phase contrast objects with micron scale structure. It is based on deriving an approximate motion model from optical flow analysis of direct reconstructions and feeding this information into the backprojection geometry, using a dynamically deformed reconstruction grids^16^. Structure and dynamics are reconstructed from the continuous stream of projection data acquired while rapidly rotating the sample, without the typical artefacts compromising conventional reconstruction. As a proof of concept demonstration for a 4d movie of a chemical reaction, we apply this approach to monitor a match in 4d, burning down after laser ignition, with sufficient spatial and temporal resolution to cover the process. While individual components of the method have been used in previous work, the combination of reconstruction steps in particular with phase contrast by free propagation, has enabled unprecedented contrast and sensitivity, high enough to uncover the dynamic change in the interior wood structure during the burn. Note that grating based phase contrast as already demonstrated for dynamic tomography^14,18^ does not achieve the high spatial resolution required for the present application.

Before we address 4d CT, we briefly recall the fundamentals of the conventional 3d CT, also in view of notational clarity. Assuming that the object scatters only weakly and diffraction within the object can be neglected, the projection images can be described based on line integrals through the object. In the simplest case of a parallel beam illumination, all line integrals traverse the object Ω parallel to the optical axis *z* and a projection is defined as^19^

1 $$P(x,y)=\int (n(x,y,z)-1){\rm{d}}z,$$

with the three-dimensional (3d) distribution *n*(*x*, *y*, *z*) of the object’s index of refraction. The wavefield Ψ_*α*_ directly behind the object is then given by

2 $${{\rm{\Psi }}}_{\alpha }(x,y)\propto \exp (ikP(x,y))\mathrm{\ ,}$$

with the wave number *k* = 2*π*/*λ* and wavelength *λ*. In a single projection, all information about the distribution of features along the optical axis is lost. Many projections from different angles are necessary to retrieve the 3d structure. According to the Fourier Slice Theorem, the Fourier transform (FT) of a projection is identical to a central slice trough the 3d FT of the object, normal to the direction of projection^20^. Thus, the 3d FT can be sampled from many projections recorded for different rotation angles of the object by adding their FT to the corresponding plane in Fourier space. An inverse 3d FT yields the structure of the object. Usually, the sample is rotated around only one axis (*γ* axis here) and projections are recorded at equidistant angular steps from *α* = 0° to *α* = 180°. In this case, any given point of the object performs a sine motion in the projections with increasing *α* and hence an *x* over *α* graph is called a sinogram. Using this sampling scheme, the sampling density increases towards the center of Fourier space and has to be weighted by a ramp filter in all planes normal to the axis of rotation. To avoid interpolation from polar to cartesian coordinates in discrete Fourier space (which requires a convolution with a sinc-kernel), the interpolation is usually performed in real space. Hence, the filtered projections are back transformed to real space, then extended into the 3d volume parallel to the original direction of projection and finally summed up to yield the reconstructed object. This most popular reconstruction method is called ‘filtered backprojection’. Other methods have also received much attention, for example algebraic reconstruction techniques (ART), which consider the problem as a set of linear equations. Notably, ART allows to derive approximative solutions from limited data (undersampled, missing wedge) by incorporating additional constraints such as positivity or a support of the object function^20,21^. These intrinsically iterative methods often come with the drawback of high computational costs but can be beneficial for time resolved tomography, since less projections are necessary. Importantly, all common tomography methods assume a static object. Motion during the measurement leads to severe artefacts like false shapes, missing features and non-closed circles as illustrated by using the ‘dynamic phantom’ shown in Fig. 1.

**Figure 2:**
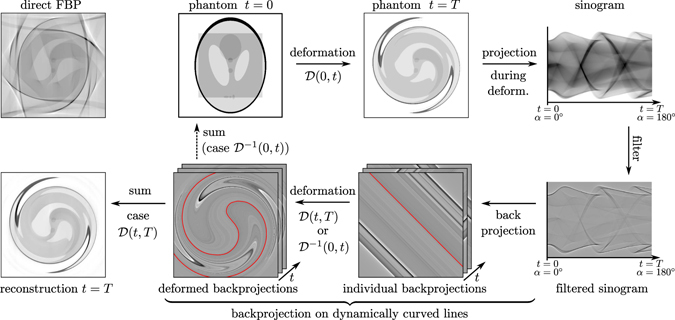
Scheme of the filtered backprojection on dynamically curved paths. The phantom is deformed as a function of time during the measurement, resulting in an inconsistent sinogram (upper row). While the conventional filtered backprojection yields a heavily hampered reconstruction (top left), the bottom row illustrates the new method, revealing a substantially improved reconstruction quality by incorporating the (known) temporal deformation. From the filtered sinogram, each individual back projection is deformed to a different degree (see text for details). The conventional straight back projection path is replaced by a dynamically curved path, as indicated by the highlighted red line. The superposition of all deformed backprojections results in a consistent representation of the phantom. As we show below, the motion model required for the dynamically curved paths can be estimated directly from the data.

Here, we demonstrate an approach for 4d (dynamic) tomography which derives high quality reconstructions from projection data that are inconsistent in the sense of conventional static tomography. Importantly, the method generalizes the usual backprojection along straight lines to dynamically curved paths, based on a motion model derived from the projections. In this way it becomes possible to compensate for general (almost arbitrary) deformations. Figure 2 illustrates the approach. For simplicity, we assume a 2d phantom. Each projection measured at the time 0 ≤ *t* ≤ *T* originates from a deformed object ${\rm{\Omega }}(t)={\mathscr{D}}(0,t)[{{\rm{\Omega }}}_{0}]$, where the operator ${\mathscr{D}}(0,t)$ describes the transformation from time 0 to time *t*. Here, Fig. 2 shows the example of a non-affine ‘swirl’ deformation. For reconstruction, each of the filtered projections is back projected into the higher dimensional (2d) reconstruction space and individually transformed by ${\mathscr{D}}(t,T)$. The superposition of all deformed backprojections yields a consistent reconstruction of the object ${\rm{\Omega }}(t=T)$ at the end of the recording. If the transformation ${\mathscr{D}}(0,t)$ can be inverted and the resulting ${{\mathscr{D}}}^{-1}(t,0)$ is applied instead of ${\mathscr{D}}(t,T)$, the reconstruction results in Ω(*t* = 0). A combination of ${\mathscr{D}}$ and ${{\mathscr{D}}}^{-1}$ allows for the reconstruction of Ω(*t*) at any given time. The backprojection and deformation steps can be merged to a single operation, i.e. a backprojection on paths which change dynamically as a function of time. In this way, the number of interpolations is reduced to that of conventional backprojection along straight paths. The scheme can also be thought of and implemented as a deformable grid, where each pixel (object feature) moves along the same path as in the original measurement, collecting the signal of all straight (untransformed) back projections consistently. As illustrated, the proposed scheme enables a dramatic increase in reconstruction quality with respect to a ‘naive’ backprojection ignoring the dynamics. For further validation, numerical simulations were performed, ranging from simple motion phantoms of linear displacements to complex non-affine types of motions and achieving excellent results. A MATLAB code for the example shown in Fig. 2 is provided as Supplemental material along with a discussion of the remaining artefacts. Note that strictly speaking, the conventional ramp-filter is no longer exactly valid for the proposed scheme of deformed paths. Future design of optimized filters could possibly be based directly on the resulting density of sampling points in Fourier space, and could be applied after the backprojection steps (as in ‘filtered layergram’). We stress, however, that the gain in reconstruction quality is considerable already for the conventional filter, even for severe deformations as shown in the example. Notwithstanding future optimizations, this allows us to already tackle substantial experimental challenges in 4d tomography, as shown below.

Before we turn to the experiment, however, we have to address the experimentally relevant situation that no dynamical model ${\mathscr{D}}(0,t)$ is known as prior information, but that the motion model has to be estimated from the data itself. In some special cases, the dynamical model could be derived from additional measurements, possibly enabled by traceable markers (feducials) in the object. However, for the general case of non-periodic and unknown motion, both structure and motion have to be derived from the projection data alone. Currently, high speed synchrotron setups are capable of recording several tomograms per second^22,23^, monitoring dynamics on the same time scale. The object is then mostly reconstructed using a sliding window approach, falsely assuming the structure to be static over half a revolution period. It is important to note, however, that while the reconstruction results suffer from significant motion artefacts which impede high quality structure analysis, approximative motion models can often be derived from such directly reconstructed data. The basic idea of the current work is to use the motion model derived from the sliding window reconstruction to implement backprojection on dynamically curved paths, as introduced for known ${\mathscr{D}}(0,t)$ above. This approach provides a significantly higher reconstruction quality, which in turn can be used to further refine the motion model. In an iterative manner, this leads to a fundamentally improved reconstruction of the entire 4d process. To demonstrate and exploit this scheme, we have performed synchrotron based phase-contrast micro-CT of a burning match, with high speed data acquisition in combination with optical-flow-based motion estimation.

**Figure 3:**
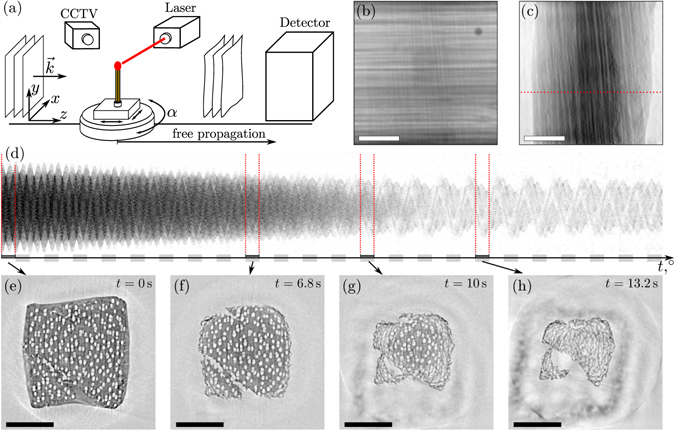
Illustration of the experiment. (**a**) Setup of the TOMCAT beamline with parallel beam illumination of the match positioned at the center of a fast rotating stage. The match was ignited by a laser system and observed by a CCTV camera. The detection system was positioned 228 mm behind the object. (**b**) A raw image recorded with an acquisition time of 1 ms, and (**c**) the same image after preprocessing and phase-retrieval (see text). (**d**) A sinogram extracted from all 18800 reconstructed projections at the position of the red dashed line. A shrinking of the structure and a loss of material is observed, accompanied by a decrease in photon absorption. The bottom row depicts tomographic reconstructions of the highlighted sinogram segments, each consisting of 401 equidistant projections. The shape, features of the wooden structure and the stages of the burning process can be clearly identified. Nevertheless, the reconstructions show motion artefacts such as ‘streaks’ and non-closed shapes in detail. Scalebars 1 mm.

The experiment was carried out at the TOMCAT beamline of the Swiss Light Source^22^. The setup is sketched in Fig. 3. A parallel beam with a photon energy of *E*_*ph*_ = 20 keV and a flux of about 10^12^ ph/s/mm^2^ was used to illuminate the object positioned in the center of a fast rotating stage. The detection system was positioned 228 mm behind the object. X-rays were converted to optical signals by a 150 *μ*m thick LAG:Ce scintillator, which was magnified by a factor of 3.73 to a high speed CCD Camera (PCO Dimax), resulting in an effective pixel size of 2.95 *μ*m. Matches made of larch wood and impregnated with paraffin wax were obtained from a local supermarket. The matches were cut to a length of approx. 8 mm and the wooden part directly under the phosphorous head was chosen as the region of interest. For the recordings, the matches were firstly aligned to fit horizontally into the field of view of the camera under all angles using position motors above the rotation stage. Then, the rotation speed was increased until the measuring speed was reached. The matches were ignited by an IR laser system^24^ and observed with an optical CCTV camera. The acquisition was started manually after the ignition flame appeared on the live monitoring screen. We did not observe an influence of the rotation on the flame and burning speed. Several burning processes were recorded with rotation speeds from 1.25 Hz up to 3.5 Hz, resulting in 2.5 up to 7 full tomograms per second. However, the duration of the measurement was limited by the internal memory of the camera system of 36 Gb. An acquisition time of 1 ms for each projection allowed the recording of the entire burning process in 18 800 images (and thus 18.8 s). At the slowest rotation speed of 1.25 Hz, 401 angles per sinogram were sufficient to reconstruct details and follow the process. Less images per sinogram led to a dramatic decrease of resolution. Thus faster rotations require shorter acquisition times, resulting in a lower signal to noise ratio not outweighed by the decrease in motion artefacts. Higher rotation speeds had the further drawback that the entire burning process could not be covered, since the maximum possible number of images was reached more quickly. Most importantly, the movies recorded at a high rotation speed in the testing phase proved that all processes observed on the resolution scale of the experiment were slow enough to be well captured by the slower recordings. Thus, we chose the measurements with a rotation speed of 1.25 Hz for our subsequent data evaluation.

A raw detector image with a size of 1200 × 1116 px is shown in Fig. 2(b). It is affected by artefacts originating from a spatially and temporally inhomogeneous illumination. These artefacts were accounted for by the usual raw data corrections. First, all images were dark-field corrected and divided by the mean of 200 flat field images of the empty beam. Due to the short acquisition time, however, flickering of the illumination was not averaged out. Notably, we observed pronounced vertical shifts and structural changes in the illumination, most probably originating from vibrations of the beamline’s monochromator, resulting in horizontal artefacts after the flat-field correction. This type of artefact is usually not visible in conventional measurements where the acquisition time is large enough to average over all vibrations. Since the undesired structures appeared to be ordered predominantly horizontally and in a narrow band of spatial frequencies, we were able to remove them by a Fourier filter derived from the flat-field images as follows: Each flat-field image was divided by the mean of the 200 empty images. Without any vibration, this results in homogeneous images, but for the present data pronounced horizontal intensity gradients appeared, resulting from the vibrations. The images were Fourier transformed and their modulus was averaged. By employing a threshold, the resulting average was transformed into a binary mask discriminating between spatial frequencies from the background artefacts and frequencies that had to be attributed to the sample. All projections of the match were filtered in Fourier space using this mask, removing only structures already appearing in the flat-field images due to vibrations. As a result, the artefacts disappeared almost completely without impairing the match’s projection. Further, we have observed a degradation of the scintillator screen, possibly due to the heat during the ignition process. After the ignition, ring-like structures appeared in all projections which were not visible in flat-field images recorded before. These rings slowly grew during the measurement. They could be removed sufficiently by averaging the projections from −180° to 180° around each projection. The ring-structure could then be isolated by an adapted high-pass-filter, and was subtracted from the corresponding image.

The resulting corrected raw-data images show very little absorption contrast of the wood cell wall. As is well known, the interaction of hard x-rays with matter can be described in continuum approximation by a complex index of refraction commonly written as *n* = 1 − *δ* + *iβ*, where *δ* accounts for the phase differences compared to the wave in vacuum and *β* for absorption^19^. For typical photon energies and materials, both *β* and *δ* are extremely small. For the present photon energy range and biomolecular matter such as cellulose, *β* is on the order of 10^−10^, while *δ* is up to three orders of magnitude larger. In order to exploit the much stronger phase shifting properties of the sample, a sufficiently high degree of spatial coherence is required, as provided by the present illumination wavefront. In combination with suitable reconstruction algorithms, this allows to improve the image contrast, enabling the fast measurements as reported here. Phase contrast cannot be measured directly, but only via indirect measurement of intensity distributions ∝|Ψ|^2^. Among the many different approaches to visualize phase shifts, only a few are suitable for time-resolved imaging. For example, many phase contrast techniques require lateral or longitudinal scanning of the object in the probing beam, notably in ptychography^25^, Talbot interferometry^26^, edge-illumination^27,28^ or speckle-based phase contrast techniques^29^. Therefore, we chose phase contrast by free propagation^30–32^ based on the self-interference of the wavefront behind the object. The phase shift information of the projection Ψ_0_ is thereby converted into measurable intensity variations. A single recording without scanning is sufficient and no additional optical elements are inserted in the optical path behind the sample, such as in Zernike based phase contrast^33^, assuring a high signal and dose efficiency. For weakly absorbing and phase-shifting objects as well as small propagation distances *d*, the detector image can be described by a linearized Transport of Intensity Equation (TIE)^19^. If the complex wavefield ${{\rm{\Psi }}}_{0}\propto \sqrt{{I}_{0}}\exp ({{\rm{\Phi }}}_{0})$ directly behind the object is expressed by amplitude $\sqrt{{I}_{0}}\propto |{{\rm{\Psi }}}_{0}|$ and phase shift ${{\rm{\Phi }}}_{0}=arg({{\rm{\Psi }}}_{0})$ the detector image at projection angle *α* is described by

3 $${I}_{\alpha ,d}(x,y)\propto {I}_{\alpha \mathrm{,0}}(x,y)(1-\frac{d}{k}[{\partial }_{x}^{2}+{\partial }_{y}^{2}]{{\rm{\Phi }}}_{\alpha \mathrm{,0}})\mathrm{.}$$

The derivatives lead to an edge enhancement of the object’s features in this so-called direct contrast regime, improving the signal quality. Phase retrieval requires the inversion of the derivatives, which can be implemented by a multiplication with ${({k}_{x}^{2}+{k}_{y}^{2})}^{-\mathrm{1/2}}$ in Fourier space acting as a low-pass filter. To avoid singularities, a regularisation parameter can be added. We have used the symmetric filter function

4 $$\mathop{F}\limits^{ \sim }({k}_{x},{k}_{y})=\frac{1-\exp [-f({k}_{x}^{2}+{k}_{y}^{2})]}{{k}_{x}^{2}+{k}_{y}^{2}},$$

with the regularisation parameter *f* = 0.001, preserving a certain degree of edge-enhancement beneficial for motion detection.

The result of performing all corrections and the phase retrieval on the exemplary projection of Fig. 3(b) is shown in Fig 3(c), along with a sinogram (Fig. 3(d)) extracted from the stack of all corrected images of a recording. It clearly shows the shrinking of the structure as well as a decrease of signal intensity, originating from the loss of mass during burning. Intervals of 401 images were selected from the sinogram to compute subsequent tomographic reconstructions. Filtered backprojections of four intervals are shown in the bottom row of Fig. 3. To achieve a well aligned vertical rotation axis in the center of the projections, which can in general not be sufficiently realized in experiments, all images were rotated by the misalignment angle (angle between the determined axis of rotation and the detector columns). Further, the distance of the determined axis of rotation to the center of all images was corrected for. Both parameters (angle and offset) were determined by comparing two projections from opposite sides, with an angular distance of 180°. For static tomography, the automatic determination of both parameters in general warrants high quality reconstructions without any sign of misalignment. Contrarily, for dynamic tomography a further refinement may be required. This is due to the fact that in a series of subsequent reconstructions even smallest deviations from the original values result in a minute sub-pixel shift of subsequent reconstructions. This misalignment is sensitively registered by the human eye as a vibration in the time series, while it would not be detectable in a single reconstruction. Therefore, both parameters have been refined manually.

**Figure 4:**
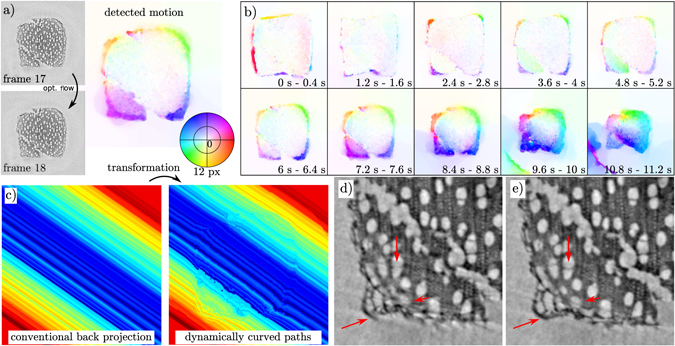
Illustration of the motion estimation for the subsequent backprojection along curved paths. Two successive reconstructions (**a**) are analyzed by an optical flow algorithm. The depicted result shows the shrinking at the borders of the match. The color indicates the direction of motion as shown by the color wheel, the saturation gives the motion amplitude with a maximum of 12 px for all images shown. The image series in (**b**) gives an overview of the dynamics of the entire burning process, showing the optical flow result for every third frame. Using the motion information, the reconstruction is the carried out by backprojection on dynamically curved paths, as illustrated in (**c**) with a magnification of the motion amplitude by the factor of 3 for better visibility. A significantly improved reconstruction quality is observed, see the fine object details resolved in (**e**), compared to the result of (**d**) a conventional direct FBP reconstruction.

As shown in Fig. 3, the described combination of corrections applied to each projection, phase retrieval and corrections of the tomographic axis resulted in a temporal series already covering the burning process remarkably well. Upon closer inspection, however, many details in particular at the moving borders are blurred by the intrinsic dynamics of the burning process, despite the relatively fast data acquisition. In the following, this issue is addressed by the backprojection on dynamically curved paths approach as introduced above. To obtain an estimation of the motion, we performed optical flow (OF) analysis^34^. Since structure and dynamics of the match vary only slowly parallel to the axis of rotation, it was sufficient to estimate the motion in the perpendicular dimensions only, considerably reducing the computational effort. In the time series, each subsequent pair of slices perpendicular to the rotation axis were compared as illustrated in Fig. 4(a). Roughly spoken, the first slice was broken up into small patches, followed by a search of the best registration within the subsequent image to obtain a map of local displacement vectors. Here, we used the powerful OF implementation from^34^. To verify the correctness of the detected motion, it was used to geometrically transform the second image to the shape of the preceeding (warping), using the so-called inverse mapping inplemented in MATLAB’s interp2 function^34^. Visual comparison of their difference showed a good agreement throughout. The motion of the entire burning process in one slice is summarized in Fig. 4(b). In the beginning, only the thin outer layer shows a shrinking behaviour. As the burning horizon moves to the center of the wooden structure, the shrinking layer of burned wood becomes thicker until the whole structure is burned. Because of the flame’s progress from the top to the bottom, the already burnt structure does not stay constant but in the end shows huge spontaneous displacement as tensions within the match change. Only in this ‘collapse’-type situation, the motion estimation failed.

Assuming a linear transformation within each tomographic interval (401 images over 180°) as a first order motion model, a reconstruction was computed as follows: For a given angle (point in time), a tomographic reconstruction from the previous and from the following 180° range of projections were reconstructed. This is particularly beneficial for streak-hampered data since the orientation of the streak motion artefacts depends on the selection of the starting angle and is the same in both reconstructions. OF analysis was then performed to derive a motion model. If, for example, the object carries out a theoretical linear global shift from *x*(*t* = 0) = −*L* to *x*(*t* = *T*) = *L* during the 360° scan, both direct reconstructions would be perturbed in similar ways. The main difference between both reconstructions would be a shift about *L* against each other, which is the same shift magnitude which the object underwent during the recording of 180°. Hence, the detected motion magnitude can be used directly without rescaling for the subsequent application of filtered backprojection on dynamically curved paths, which is used to ‘sharpen’ the reconstruction of the second set of projections to the given point in time. Figure 4(c) shows an exemplary backprojection with the deformation enlarged by a factor of 10 for better visibility. With this approach, the motion artefacts were significantly suppressed, and the reconstruction quality was significantly improved, compare for example the slices shown in Fig. 4(d) (without correction) and (e) (with correction). Both images depict an enlarged region of the object, reconstructed from the same projections. The direct reconstruction in (d) shows artefacts especially at the outer border of the match including non-closed disrupted structures and clearly visible streaks. In the motion based reconstruction, these artefacts are considerably reduced. New details in the wooden structure become visible as indicated. At this point, the entire process of motion estimation and correction can be iterated with the higher quality reconstructions. However, for the match presented here, a single iteration appeared to be sufficient.

**Figure 5:**
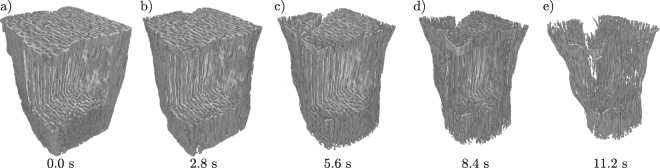
Rendered 3d structure of the burning match at different times. For the corresponding movie see Supplemental data.

Based on the proposed dynamic backprojection approach, the dynamics of the burning process can now be investigated with high image quality and at high spatial and temporal resolution. The 4d nature and amount of analyzable data is illustrated in Fig. 5, showing the rendered 3d structure at 5 different times of the process. A high quality movie of the dynamics is available as online material, including single slices as well as the entire rendered structure.

In summary, we have demonstrated a novel combination of techniques for 4d tomography i.e. backprojection along dynamically curved paths and optical flow analysis. This optimized reconstruction from the projection data stream provides ‘material movies’ without the typical motion artefacts and at spatial and temporal resolution sufficient to monitor micron scale structural changes during combustion. As a proof-of-concept we have studied the burning process of a match in 4d, which after a hundred years of using matches in everyday life has certainly never before been studied in 4d. Choosing wood as a material, and a simple burning as a reaction, the example also covers one of the first materials and material processes used by mankind. To record the changes of the cellular wood structure (tracheids with cell walls and lumen) during the burn process, phase contrast were necessary, since is much more sensitive to softer materials and smaller structures.

The information contained in the 4d reconstructions is ‘bulky’, and cannot be visualized well by 2d prints. To this end, we refer the reader to the Supporting material, including movies and rendering. The supporting data also includes the analysis of some elemental observables in the burning process, notably the mass, volume and interior surface as a function of time. Aside from the algorithm, several experimental and instrumental aspects were of particular importance for this work, notably the fast cameras. Regarding detection and data transfer, the GigaFRoST technology at the Swiss Light Source will provide a further advancement, with continuous recording at a rate of up to 8 GB/s^23^. Aside from the beamline optics, instrumentation and recording technology, we want to stress that phase retrieval was instrumental in this work to reach quantitative gray values, and thereby facilitate the optical flow analysis. Furthermore, the raw data treatment and filtering of illumination artefacts was also a necessary step to reach the level of data quality where the phase retrieval and optical flow analysis could be put to operation. Note that the role of empty beam division and aberrations in the illumination wavefront has previously been addressed in ref.^35^. Several improvements of the present schemes can be directly envisioned: (i) Curved path back projection and the motion estimate can be iterated, as mentioned above. (ii) The motion estimate can be carried out beyond linear interpolation. For example, the acceleration of the motion can be investigated by starting the calculation of the optical flow at a slightly different angle and comparing the sets of optical flow images. (iii) The concept of dynamically curved path can be extended from 2d slices to 3d volumes. (iv) Finally, the concept of backprojection along dynamically curved paths can be combined with the regularized models of dynamic CT, based on prior information.

Using this combination of phase contrast recordings, motion estimates based on optical flow, and subsequent backprojection along dynamically curved paths based on the motion model obtained from the data it should also become possible in future to achieve the spatial and temporal resolution required for the observation of material processes on the nanoscale. To this end, the parallel beam illumination used here has to be replaced by a cone beam geometry with nanoscale secondary focal spot size^36^.

## Electronic supplementary material

Supplementary txt file

Supplementary Information

Supplementary Video
