# Publisher Correction: Four dimensional material movies: High speed phase-contrast tomography by backprojection along dynamically curved paths

**DOI:** 10.1038/s41598-018-23449-5

**Published:** 2018-03-22

**Authors:** A. Ruhlandt, M. Töpperwien, M. Krenkel, R. Mokso, T. Salditt

**Affiliations:** 1 Institut für Röntgenphysik, Universität Göttingen, Friedrich-Hund-Platz 1, Göttingen, Germany; 2 Swiss Light Source, Paul Scherrer Institute, 5232 Villigen, Switzerland

Correction to: *Scientific Reports* 10.1038/s41598-017-06333-6, published online 26 July 2017

The supplementary video showing a rendering of the structural changes of the wooden structure of the matchstick while burning down was omitted from the original version of this Article. In addition, the original PDF version of this Article contained a corrupted version of Figure 5.

These errors have now been corrected in the PDF and HTML versions of the Article.

