# Supplementary material for: Four dimensional material movies: High speed phase-contrast tomography by backprojection along dynamically curved paths
Source: Sci Rep. 2017 Jul 26;7:6487. doi: 10.1038/s41598-017-06333-6 (PMC5529574; doi:10.1038/s41598-017-06333-6)
Supplement: Supplementary file 2 — Supplementary Information [file 41598_2017_6333_MOESM2_ESM.pdf]

Four dimensional material movies: High speed phase-contrast tomography by backprojection along dynamically curved paths – Online Material

A. Ruhlandt, M. Töpperwien, M. Krenkel, and T. Salditt*
Institut für Röntgenphysik, Georg-August-Universität Göttingen, Friedrich-Hund-Platz 1, Göttingen, Germany

R. Mokso
Swiss Light Source, Paul Scherrer Institute, 5232 Villigen, Switzerland and Max IV Laboratory, Lund University, Fotongatan 2, 225 94, Lund, Sweden
(Dated: March 31, 2017)

Along with this document, you find a video and a MATLAB file. The video shows a rendering of the structural changes of the wooden structure of the matchstick while burning down. The MATLAB script *motion_artefacts.m* contains the simulation of a sinogram of a moving object and the reconstruction depicted in Fig. 2 in the main document. In the script, reconstructed phantoms can be analysed and compared with the originals. This reveals for example the deviations shown in Fig. 1, which originate from interpolation artefacts and the fact, that the conventional ramp-filter is no longer exactly valid for the proposed scheme of deformed paths. However, despite the severe deformation the features of the object are clearly reconstructed, while a direct reconstruction of the sinogram leads to completely useless results. For further details we refer the reader to the script.

The four dimensional nature of the reconstructions allows for extracting previously unaccessible observables. As an example, the number of 'unburned' voxels with an absorption value above a threshold can be compared with the number of exposed surfaces of these voxels, giving a measure of the compactness of the structure. For the bottom half of the reconstructed volumes, which are not disturbed by the phosphorus head entering the volume during the burning process, both values have been plotted in Fig. 2. The ratio of volume over area decreases over time, indicating that the matchstick becomes more porous. The general trend of both volume and area, i.e. the change in steepness of the curves, shows a similar progress of the burning process as the x-ray transmission plotted in Fig. 3.

Fig. 4 compares two reconstruction methods of a single slice of the match. On the left side the result of a common filtered back-projection is shown. The right side depits the result of a Simultaneous Iterative Reconstruction Technique (SIRT) after 200 iterations from the same data. Notice that a sigle SIRT iteration needs arround twice the computation time as the FBP, since each steps requires a full backprojection and reprojection of the object. Nevertheless, the reconstruction using SIRT lacks many actual details compared to the FBP result. For this comparison we relied on the SIRT implementation in the ASTRA-toolbox [1, 2].

[1] van Aarle, W. *et al.* Fast and Flexible X-ray Tomography Using the ASTRA Toolbox. *Optics Express* **24**, 25129–25147 (2016). http://dx.doi.org/10.1364/OE.24.025129.

[2] van Aarle, W. *et al.* The ASTRA Toolbox: A platform for advanced algorithm development in electron tomography. *Ultramicroscopy* **157**, 35–47 (2015). http://dx.doi.org/10.1016/j.ultramic.2015.05.002.

* tsaldit@gwdg.de

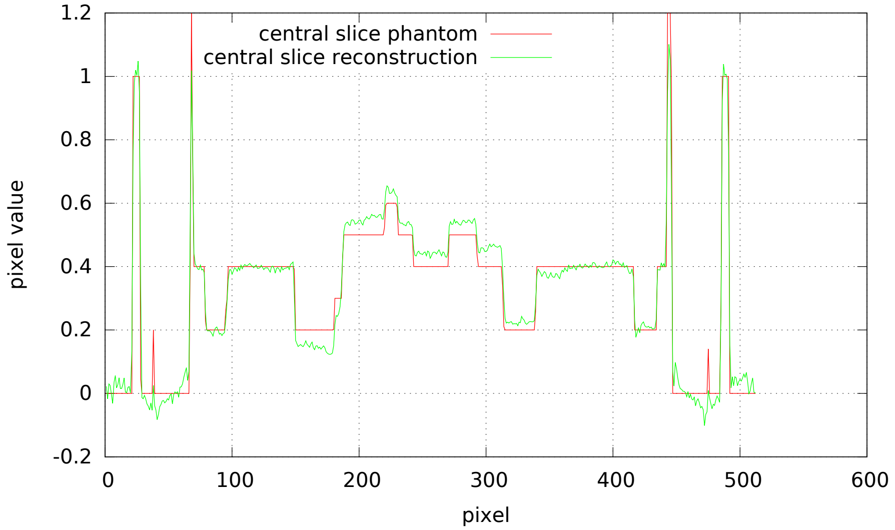

FIG. 1. Comparison of the reconstruction in the script `motion_artefacts.m` with the phantom. The features of the phantom are clearly reproduced but distorted by interpolation artefacts.

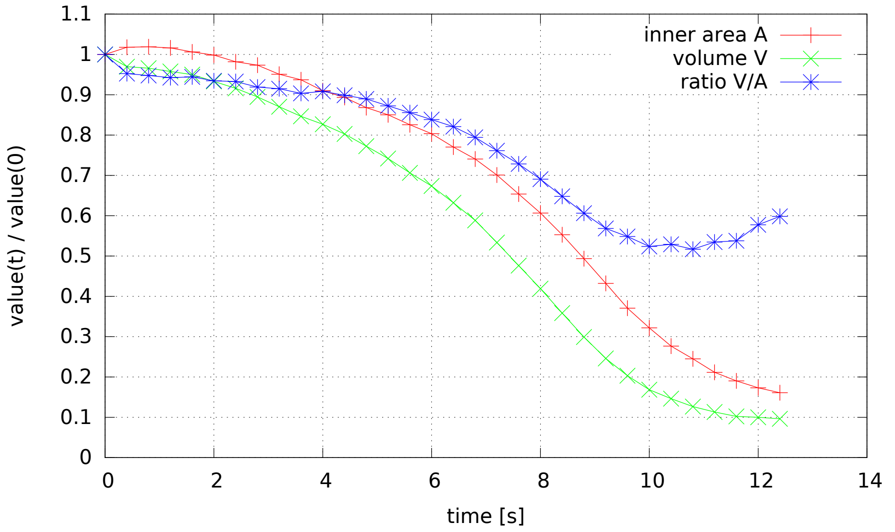

FIG. 2. Temporal evolution of the volume V and inner area A of unburnt voxels, as well as the ratio of both.

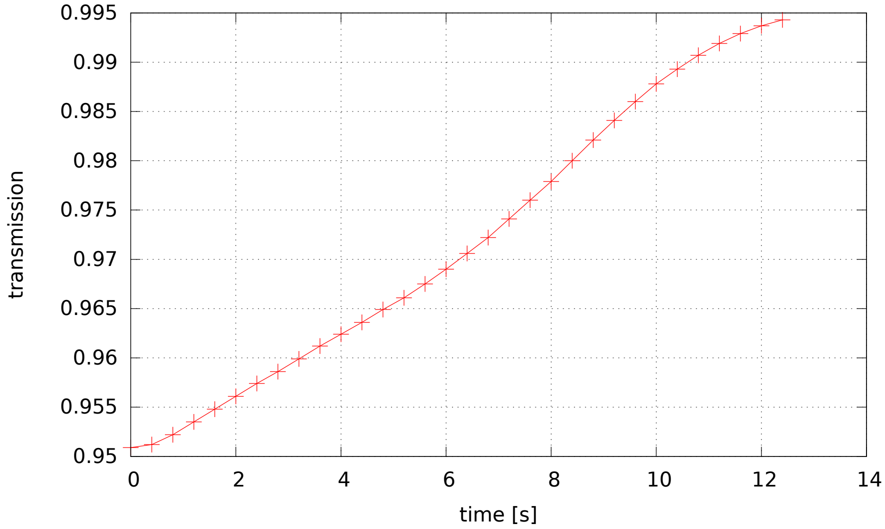

FIG. 3. Temporal evolution of the X-ray transmission of the object. Regions of smaller slope (e.g. from 0 s to 5 s and from 10 s to 13 s) correspond to regions of smaller slope in Fig. 2. The same is true for regions with steeper slope (from 7 s to 10 s).

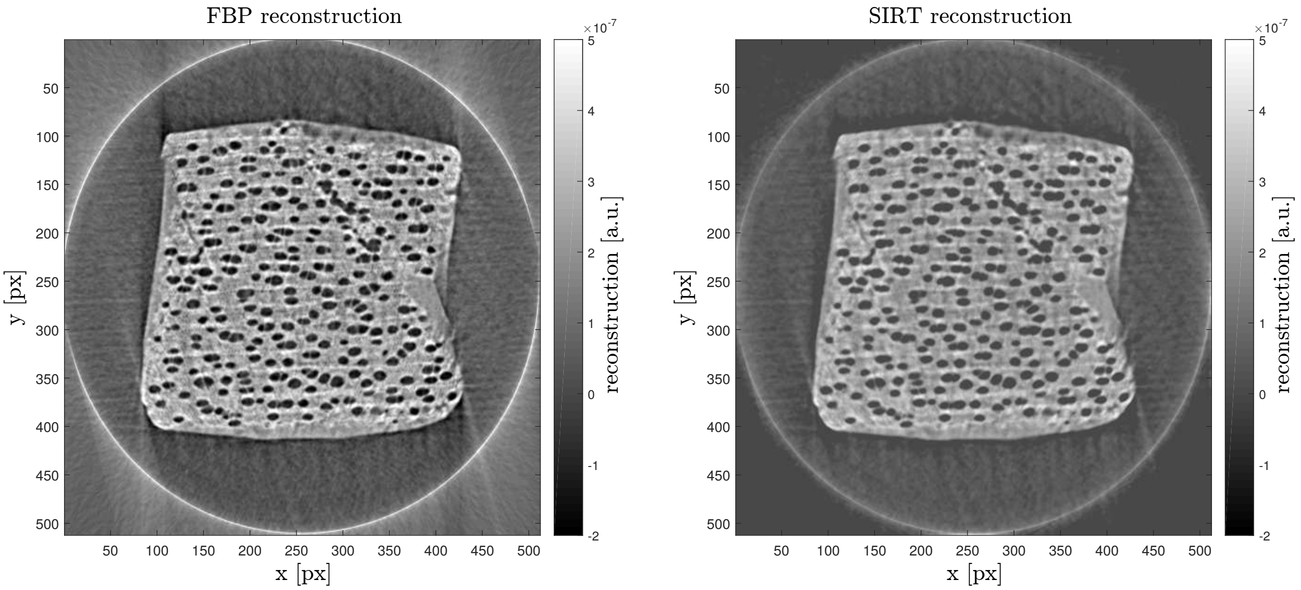

FIG. 4. Comparison of reconstruction methods. Left: Filtered Back-Projection (FBP), right: Simultaneous Iterative Reconstruction Technique (SIRT) after 200 iterations.
